# Multiple, active-offer referrals for HIV pre-exposure prophylaxis by nurses yields high uptake among gay, bisexual, and other men who have sex with men

**DOI:** 10.1177/09564624231220084

**Published:** 2023-12-06

**Authors:** Lauren Orser, Patrick O’Byrne

**Affiliations:** School of Nursing, 6363 University of Ottawa, Ottawa, ON, Canada

**Keywords:** HIV prevention, preexposure prophylaxis, risk factors, men who have sex with men

## Abstract

**Introduction:**

Current Canadian guidelines focus on indications and uptake of preexposure prophylaxis (PrEP) among groups at-risk for HIV, such as gay, bisexual, and men who have sex with men (GBM). Less, however, is known about the outcomes of PrEP offers. This study presents on the responses of GBM to multiple offers for PrEP.

**Methods:**

In Ottawa, Canada, we instituted Canada’s first nurse-led PrEP program, pre-exposure prophylaxis by nurses (PrEP-RN), where nurses offered PrEP referrals to persons with indicators for HIV. Responses to offers from nurses and HIV diagnoses were recorded and assessed for multiple occurrences based on responses. Descriptive analyses were used to report frequencies and percentages of findings and chi-square analyses were conducted to determine significance based on HIV risk for those who accepted versus declined PrEP

**Results:**

Over a 4-year period, 644 PrEP offers were made to 236 unique patients, all of whom were GMB, the majority of whom identified as male. Of the eligible patients, 50.8% accepted and 50.0% declined after multiple offers. Seven trajectories were identified in terms of reasons for accepting or declining PrEP. PrEP referrals made based on clinical guidelines and to those who had changes in risk factors over time were significantly correlated with PrEP acceptance. We noted five HIV diagnoses, all of which were among GBM who declined PrEP at least once.

**Conclusions:**

Based on these findings, it appears multiple PrEP may yield increased PrEP acceptance among a sample of GBM.

## Introduction

Canadian clinical guidelines detail how and to whom clinicians should provide HIV preexposure prophylaxis (PrEP), including information about which medications to prescribe, plus the types and frequencies of testing to complete to ensure safe administration.^1^ These guidelines also detail indicator conditions for PrEP, which identify those who are at highest risk for HIV seroconversion, including gay, bisexual, and other men who have sex with men (GBM) who engage in condomless anal sex and individuals who have exposure to a partner with transmissible HIV.^1^ Table 1 shows the indicator conditions often associated with elevated risk for HIV acquisition. Other literature describes the outcomes and utility of expanding PrEP delivery from physicians to nurses, as well as how public health units can incorporate PrEP into their work.^2,3^

**Table 1.** High-risk indicators for PrEP referrals.

High-risk criteria
Clinical guidelines:
• Contact of person with transmissible HIV
• Infectious syphilis diagnosis
• Rectal chlamydia
• Rectal gonorrhea diagnosis
• LGV diagnosis
• Post-exposure prophylaxis (PEP) use
Clinical judgment
• Based on reported risk practices

Inherent within much of the foregoing literature on and about PrEP are assumptions that patients are inherently driven to use this intervention, and that they would therefore naturally accept PrEP were it offered to them^4,5^; real-world data, however, suggest otherwise. For example, the nurse-led PrEP-RN clinic in Ottawa, Canada identified that over half of patients with indicator conditions for PrEP declined this intervention when it was offered to them for free.^2^ Simply knowing how to prescribe PrEP and who would benefit from it thus appears to be insufficient to maximize PrEP uptake. We also need to know about who declines PrEP and how to adapt our approach to PrEP offers to improve PrEP uptake among individuals most affected by HIV. To that end, we sought to evaluate the effect of offering PrEP at multiple instances to GBM at elevated risk for HIV infection to determine if repeated discussions yielded changes in responses or HIV risk over time.

To address this question, we report on the responses for PrEP offers to persons we offered a referral to more than once and the incidental HIV diagnoses identified among those who received multiple offers for PrEP.

## Methods

### The intervention: PrEP-RN

Pre-exposure prophylaxis by nurses was implemented on August 6, 2018 with the intention of (1) identifying individuals at elevated risk of HIV acquisition based on established risk correlates, (2) providing information on HIV prevention options to these persons, and (3) offering this same group immediate referrals to PrEP care. These referrals were made by public health nurses completing follow-up for reportable sexually transmitted infections through the public health department (including, infectious syphilis, chlamydia, and gonorrhea), as well as nurses and other providers in a single sexual health clinic in downtown Ottawa. Any person who fulfilled the risk criteria noted in Table 1, was offered a PrEP referral either to our PrEP-RN clinic for expedited initiation or a local PrEP provider. As well, individuals who belonged to HIV priority groups (e.g., GBM), but did not meet the high-risk criteria were offered a referral to a local PrEP provider.

The PrEP-RN clinic operated within our local sexual health clinic. The primary aim of this clinic was to provide a site for rapid PrEP initiation for individuals who were identified as being at elevated risk for HIV acquisition. Once stabilized on PrEP, these patients were referred to external providers for continuation within 1 year to allow for other, at-risk individuals to access services via PrEP-RN. The Ontario HIV Treatment Network provided funding to pay for PrEP medication (emtricitabine/tenofovir disoproxil fumarate) for patients who did not have medication insurance.

### Data collection and analysis

Each time PrEP was offered, nurses completed a referral sheet, which recorded whether patients met the high-risk criteria for PrEP – and if so, which, and the response to the offer. Patients who did not meet the high-risk criteria in Table 1, but belonged to a group with elevated HIV prevalence (e.g., GBM) were still offered a PrEP referral, but were classified as lower risk. Data from the referral sheets were logged in an Excel spreadsheet, which captured patients’ chart numbers, sex, age, sexual practices (sex of sex partners), criteria for PrEP referral, and response to PrEP offer. Ethnicity and race data was not collected in the documentation of PrEP offers as these were not part of regulatory data collection for sexually transmitted infection follow-up in Ontario. We also recorded any new HIV diagnoses among persons who we had offered PrEP to.

Through Excel, we identified repeating chart numbers of patients who accepted, declined, or were ineligible for, PrEP. Data were collected over a 4-year period – from August 6, 2018 to August 5, 2022. To be included in this review, participants had to have been offered PrEP more than once. A separate log was used to capture the date of each encounter, patients’ response, if they met the high-risk criteria, and if risk factors changed over time (e.g., from low to high-risk or if new high-risk indicators were identified).

Data were analyzed descriptively to report on frequencies and averages of PrEP offers and HIV diagnosis rates. We also completed chi-square analyses looking at those who accepted or declined PrEP who were (1) offered PrEP based on clinical guidelines (contact of person with transmissible HIV, recent STI diagnosis, or PEP use) compared to those who were offered based on clinical judgement; (2) considered high-risk (based on all criteria in Table 1) compared to those where not high-risk; and (3) who had a change in HIV related risk practices over time compared to those who did not.

### Ethics

The University of Ottawa research ethics boards approved this study (H-04-18-533).

## Results

From August 6, 2018 to August 5, 2022, we offered PrEP to 2014 eligible persons, of whom, 49% (*n* = 985/2014) accepted a referral. From this total sample, 39% (*n* = 778/2014) fulfilled the criteria in Table 1, of whom 44% (*n* = 339/778) accepted PrEP and 56% (*n* = 439/778) declined PrEP.

In reviewing multiple offers, during the same study period 644 offers for PrEP were made to 274 unique GBM patients. Thirty-eight of these patients were either previously established on PrEP, but offered a referral at the point of STI assessment or diagnosis or were offered PrEP once and found to be in care during subsequent offers. These 38 patients were excluded from the analysis, leaving a total of 236 patients who received multiple offers for PrEP. Five patients were found to be HIV-positive during the study period, all of whom had declined PrEP at least once. In reviewing the outcomes associated with multiple offers, we identified that participants followed seven trajectories, which are summarized in Table 2.

**Table 2.** Trajectories for PrEP referrals.

Trajectory	Ultimate outcome	Pathway	HIV diagnoses
1	Accepted (*n* = 120) 2.5 offers	Declined then accepted (*n* = 54)	0
2		Accepted, no initiation, then accepted (*n* = 49)	
3		Used before, discontinued, then accepted (*n* = 17)	
4		Declined then started elsewhere (*n* = 5)	
5	Declined (*n* = 118) 2.4 offers	Declined all offers (*n* = 94)	5
6		Accepted, no initiation, declined further offers (*n* = 20)	
7		Used before, discontinued, declined to restart (*n* = 8)	

The majority (*n* = 234) of eligible patients identified as cis-male and were an average age of 30 years old (range: 17 to 68). A full 228 patients reported having only same sex partners and eight reported engaging in sex with same and opposite sex partners.

### Multiple PrEP referrals–accepted

**Figure 1.**
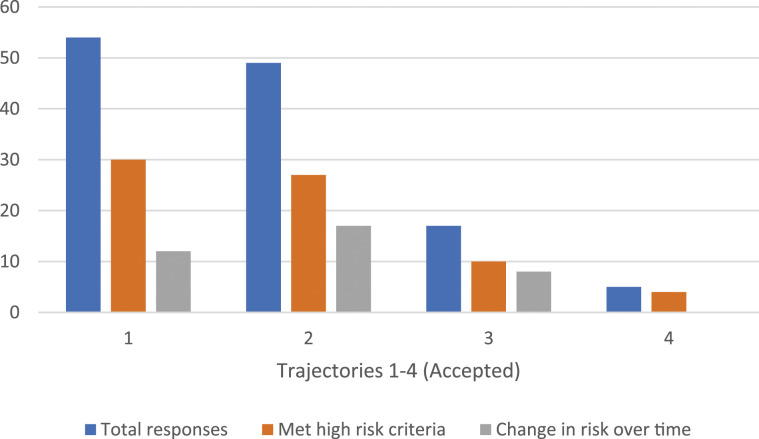
PrEP Accepted, per trajectory.

Of patients who received multiple offers for PrEP, 50.8% (*n* = 120/236) accepted a referral, of whom 62.5% (*n* = 75/120) met our high-risk criteria (Table 1). The average number of offers for patients to accept PrEP was 2.5 (range: 2–6 offers). In terms of reasons for accepting after repeated discussions, we identified four unique trajectories (Table 2, Figure 1). The most common pathway was that patients accepted a referral at multiple visits. In this case, patients initially accepted, but did not initiate PrEP, so when it was offered again, they accepted a new referral. Multiple acceptances were noted among 45% (*n* = 54/120) of patients who accepted a referral, at an average of 2.5 offers until they actually initiated PrEP. A full 55% (*n* = 30/54) met the high-risk criteria, half of whom had multiple HIV risk indicators identified over the study period. An additional 12 had a change in HIV risk factors over time per Table 1. The next most common pathway was that patients initially declined PrEP but accepted it at a later visit, which occurred among 40.8% (*n* = 49/120) of patients who accepted after an average of 2.5 offers. Of those who initially declined PrEP, 55.1% (*n* = 27/49) met the high-risk criteria for referral. Of those 49 patients, 17 had subsequent or new high-risk indicators identified over the study period as indicated in Table 1.

Less common pathways for accepting PrEP after multiple offers were identified among 14.1% (*n* = 17/120) of patients. 12 GBM had previously used and discontinued PrEP, and accepted nurses’ offer to re-start PrEP at an average of 2.5 times. 10 of these 12 patients were identified as high-risk for HIV acquisition at the time of initial offer and eight had change in risk practices or new high-risk indicators. The last pathway for accepting PrEP was patients who had previously declined referrals but had started PrEP elsewhere. These participants did not receive referral for care through PrEP-RN, but independently initiated PrEP through another health clinic; this occurred among five persons after an average of 2.7 offers. Of those who started PrEP elsewhere, four of five met the high-risk criteria and accepted PrEP after an average of 2.9 discussions.

For HIV diagnoses, to the best of our knowledge, no patient who accepted PrEP after multiple offers was found to have been diagnosed with HIV during the 4-year study period.

In summary, of the 120 patients who accepted a referral for PrEP after multiple offers (average 2.5 offers), 62% (*n* = 75/120) met our high-risk criteria for HIV acquisition per Table 1. Using these criteria, the average number of offers made for PrEP referral to higher-risk individuals increased marginally to 2.6 attempts. Among those who accepted PrEP, 72% (*n* = 54/75) were found to have a change in risk practices over the study period related to a new or subsequent STI diagnosis or sexual risk exposure(s) as per the criteria noted in Table 1.

### Multiple PrEP referrals–declined

**Figure 2.**
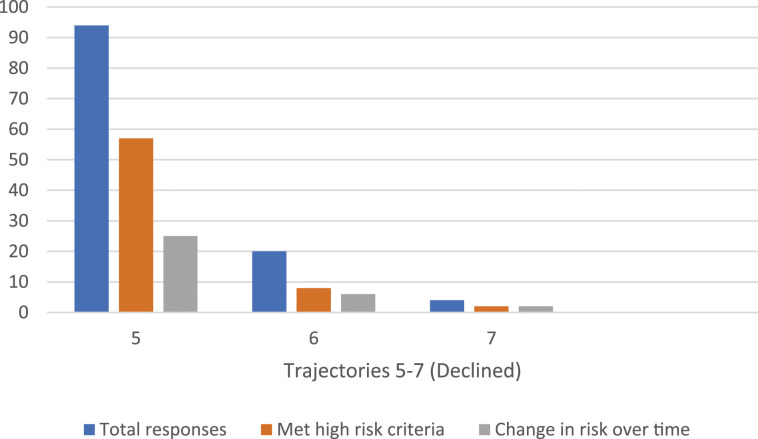
PrEP Declined, per trajectory.

Of the total patients who were offered PrEP, 50.0% (*n* = 118/236) declined multiple times after an average of 2.4 offers. We identified three main trajectories for those who ultimately opted not to use PrEP (Table 2, Figure 2). The most common pathway was that patients declined multiple offers for PrEP, which occurred in 79.7% (*n* = 94/118) of cases after an average of 2.3 offers. Of those who consistently declined PrEP, 48.3% (*n* = 57/118) met the high-risk criteria, of whom, 25 had multiple or new high-risk HIV indicators identified during the study period per Table 1.

The next most common pathway was that patients initially accepted a referral for PrEP, but never initiated it and declined subsequent offers for re-referral. This group accounted for 16.9% (*n* = 20/118) of multiple declined offers after an average of 2.9 discussions. Of the individuals who changed their minds about re-starting PrEP, 40% (*n* = 8/20) were identified as high-risk for HIV acquisition based on the referral criteria in Table 1 and 30% (*n* = 6/20) had a change in HIV-related risks over time, having had new high-risk indicator identified over the study period. Lastly, fewer than five patients had previously used PrEP and declined an offer to re-start. Half of these individuals met the high-risk criteria and half had changes in risk practices over time.

For HIV seroconversions, five patients who ultimately declined PrEP were diagnosed with HIV during the study; all fulfilled our high-risk criteria per Table 1. For the entire sample who met our high-risk criteria (including those who received multiple offers), these five diagnoses yielded an HIV seroconversion rate of 0.6% (*n* = 5/778), or a rate of one new HIV diagnosis per 156 persons who met the high-risk criteria for PrEP use. Among our entire sample who declined PrEP (which is the group in which all diagnoses were identified), the seroconversion rate was 1.1% (*n* = 5/439), for a rate of one new HIV diagnosis per 88 persons.

In summary, of the 118 patients who declined a referral for PrEP after multiple offers (average 2.4 offers), 44.1% (*n* = 52/118) met our high-risk criteria for HIV acquisition. Using these criteria, the average number of offers made for PrEP to higher-risk individuals was 2.6 attempts. Among those who declined PrEP, 21.2% (*n* = 25/118) were identified as having a change in HIV risk factors over the course of the study period.

### Analysis

Those who ultimately accepted (vs declined) PrEP were more likely to have been offered PrEP due to fulfilling clinical guidelines compared those who were offered based on clinical judgement (63% vs 46%) (chi-square 6.69, *p* = .009), and were more likely to have reported a change in risk practices over time compared to those with no change in risk practices (78% vs 32%) (chi-square 42.71, *p* < .01). There was no difference in ultimate acceptance versus decline of PrEP based on if persons had a high-risk indication based on clinical guidelines or clinical judgment (per Table 1) compared to those who did not meet any of the high-risk indicators, but were offered PrEP (chi-square 3.27, *p* = .07).

## Discussion

Our descriptive analysis of 236 GBM patients who were offered PrEP multiple times found that half accepted a referral and half declined a referral. The average number of referrals made was consistent between these two groups at 2.5 offers and 2.4 offers, respectively; this increased slightly to 2.6 offers (for both groups) when accounting for offers made to GBM who met our high-risk criteria. While the overall number of referrals made was similar, there were notable differences in risk practices between the accepted and declined groups over time. Specifically, 63% of patients who accepted PrEP were found to have new HIV risk factors over the study period (e.g., new STI diagnosis, contact of person with transmissible HIV, PEP use), whereas only 48% of patients who declined PrEP had a change in HIV risk factors. In addition, five patients who were found to be HIV-positive during the study period had declined multiple PrEP referrals.

Considering that, to the best of our knowledge, this is the first research to explore multiple offers for PrEP, this paper adds some useful insights on the utility of this intervention for GBM with high-risk indicators for HIV acquisition. Despite its established efficacy, current literature indicates PrEP use among GBM is affected by misconception, stigma, and lack of accessibility.^6–9^ Misconceptions commonly relate to beliefs that PrEP is harmful to the body (i.e., nephrotoxic) and to concerns regarding the safety of long-term, continuous medication use.^6^ In addition, some GBM believe that the risk threshold required for PrEP is high (e.g., for those who have multiple sexual partners) or that PrEP promotes condomless sex.^7–9^ Feelings of stigmatization have also been identified as reasons not to use PrEP, which relate to perceptions that PrEP is indicated for an alleged high-risk *other*.^8^ That half of all persons to whom we offered PrEP declined signals that further work is required here. However, that some persons did opt to initiate PrEP after multiple offers suggests this could be a useful strategy to increase uptake.

It is also relevant to acknowledge that the changes in responses to PrEP offers could be an artefact of time between these offers, during which, patients could reflect on their perceived need for PrEP. While the majority who declined PrEP appeared fixed in their decision, these discussions did provide an opportunity for nurses to provide counselling on PrEP for future consideration. Moreover, most individuals who accepted a referral either did so multiple times or had a change from their initial response (declined to accepted) indicating that repeat offers at every clinical interaction may be beneficial to engage patients in HIV prevention care.

Repeated discussions about prevention services in clinical settings is not a novel concept. Research on smoking cessation has established that brief (1–3 min) or very brief (30 s) advice, including assessing interest in reducing/stopping use and providing supports, had a significant impact on rates of smoking cessation.^10,11^ The same principles of smoking cessation could be applied to discussions of HIV prevention strategies for members of the groups most affected by HIV, such as GBM. Using brief or very brief interventions,^10,11^ nurses could provide advice (less than 3 min) regarding PrEP and assess patients interest in a referral at every clinical encounter, particularly when the reason for visit is related to sexual health.

Following the 3 A’s approach^11^ (Ask, Advise, Assist) used for smoking cessation, nurses would: (1) ‘Ask’ if patients had heard about, or considered, using PrEP; (2) ‘Advise’ patients on the risks, benefits, and options regarding PrEP; and (3) ‘Assist’ patients by making a PrEP referral for those who express interest in one or providing resources for those who are pre-contemplative.^11^ Repeating these discussions at subsequent visits also allows patients to consider the information provided and offer made. When PrEP is declined (regardless of the number of offers made), nurses should also provide brief information on other HIV prevention options, such as PEP and routine HIV screening.^2^ This simple adaption based well-established interventions could yield increased contemplation for, and acceptance of PrEP, as was the case in our sample of GBM.

The number of diagnoses in this study support our recommendations to offer PrEP multiple times to persons with risk factors for HIV, and to consider doing so using the 3 A’s approach^11^ as guidance. With one out of every 88 persons who declined our offers for PrEP being diagnosed with HIV during this study, ongoing prevention efforts – including multiple offers for PrEP – are not just appropriate, but required. Further supporting our recommendations for multiple offers is that, in Ottawa, the number of new HIV diagnoses among GBM decreased by 82% during the study period, yielding only 29 new HIV diagnoses among GBM locally during the entirety of this research.^12^ This signals that, although the number need-to-treat (NNT) of 1:88 is lower than the NNT of 1:50 identified in most of the literature,^13^ our rate occurred in a context of significant decreases in new HIV diagnoses among GBM and these five new diagnoses still accounted for 17% (*n* = 5/29) of the total HIV diagnoses among GBM during our study. Therefore, as the NNT is influenced by incidence, it is unsurprising that our NNT was lower than, although still higher than identified for most lower risk groups.^13^

## Limitations

This research has several limitations. First, less than five participants in this sample identified as female or trans, indicating that multiple offers appear to have been primarily limited to GBM. Second, ethnicity-based data were not collected for participants outside of those seen for care within the PrEP-RN clinic. As a result, we are unable to draw conclusions about the effectiveness of multiple offers to specific gender or racial/ethnic groups. Finally, some referral forms were received without an associated chart number, so we were unable to link unique participants together, which could have affected the overall rate of PrEP referrals made (i.e., numbers presented may be lower than actually occurred).

## Conclusion

In conclusion, our examination of PrEP referrals made to a cohort of GBM yielded high uptake in acceptance when these offers were made over multiple encounters with nurses and other providers. While approximately half of our patients declined a PrEP referral after multiple offers, the other half accepted. Considering that 43% of those who accepted PrEP had initially declined, brief discussions regarding, and repeated offers for, PrEP seems to have positively affected uptake in this study. That all HIV seroconversions in the study sample occurred among patients who declined PrEP signals that repeated discussions about HIV prevention options is prudent to limit ongoing HIV transmission to priority populations, such GBM. Providing active-offer PrEP referrals using the adapted ‘3 A’ approach for HIV prevention could, however, be a useful starting point to help reduce HIV incidence.
